# *Porphyromonas gingivalis* gingipain potentially activates influenza A virus infectivity through proteolytic cleavage of viral hemagglutinin

**DOI:** 10.1016/j.jbc.2025.108166

**Published:** 2025-01-08

**Authors:** Noriaki Kamio, Marni E. Cueno, Asako Takagi, Kenichi Imai

**Affiliations:** Department of Microbiology and Immunology, Nihon University School of Dentistry, Tokyo, Japan

**Keywords:** influenza virus, oral bacteria, *Porphyromonas gingivalis*, hemagglutinin, gingipain

## Abstract

Influenza is a worldwide health problem that causes significant morbidity and mortality among the elderly; therefore, its prevention is important. During influenza virus infection, the cleavage of hemagglutinin (HA) is essential for the virus to enter host cells. Influenza virus-bacteria interactions influence the pathogenicity of infections, and specific bacteria contribute to the severity of the disease by participating in HA cleavage. Poor oral hygiene and the presence of oral bacteria are associated with influenza. *Porphyromonas gingivalis*, a periodontopathic bacterium, is particularly associated with influenza; however, the underlying mechanisms remain unclear. In the present study, we observed *P. gingivalis* culture supernatant promoted viral release and cell-to-cell spread of the infection. Further investigation revealed that the supernatant contained cleaved HA. Therefore, we focused on gingipains (Rgp and Kgp) which are trypsin-like proteases produced by *P. gingivalis*. We determined that the Rgp inhibitor inhibited both HA cleavage and the increase in virus release associated with the *P. gingivalis* culture supernatant, whereas such effects were not observed with the Kgp inhibitor. In addition, Rgp-deficient *P. gingivalis* culture supernatant failed to cleave HA, enhance virus spread, or increase virus release. In contrast, Kgp-deficient *P. gingivalis* culture supernatant cleaved HA and promoted infection. These results indicated that *P. gingivalis*-secreted Rgp has the potential to activate influenza virus infectivity through HA cleavage, suggesting that understanding the effects of *P. gingivalis* on influenza virus infection will contribute to the establishment of influenza prevention measures.

Influenza viral infections, including seasonal and pandemic outbreaks, are an important public health problem worldwide, causing significant morbidity and mortality (1, 2, 3). Annual seasonal influenza epidemics result in approximately one billion infections, 3 to 5 million cases of severe illness among immunocompromised individuals (such as the elderly and very young), and 300,000 to 500,000 deaths worldwide (2). In 2009, the first influenza pandemic of the 21st century was caused by a novel influenza A virus, A(H1N1)pdm09, which resulted in more than 284,000 deaths worldwide (4). Vaccination provides important protection from influenza and its potential complications (5, 6). However, significant variations in influenza vaccine effectiveness have been observed across different influenza virus types and age groups (7, 8, 9). Therefore, developing various influenza prevention methods other than vaccines is also important.

The influenza A virus possesses two surface spike glycoproteins, hemagglutinin (HA) and neuraminidase (NA), which play important roles in adsorption and release during viral proliferation (10). During the initial stages of infection, HA is critical for binding to sialic acid on the host cell surface and for fusion with both viral and endosomal membranes. HA is synthesized as a single polypeptide chain, HA0, which is posttranslationally cleaved at the arginine (Arg [R]) residue by host proteases into two subunits, HA1 and HA2 (11). This cleavage stage is essential for viral entry into the host cell through receptor-mediated endocytosis, and HA plays an important role in influenza virus pathogenicity (12). Some soluble cellular proteases, such as tryptase Clara and mini-plasmin, cleave HA (13, 14). In addition, rats treated with tryptase Clara inhibitors and infected with influenza virus have been shown to exhibit reduced virus titers in the lungs (15). Accordingly, soluble cellular proteases play an important role in influenza virus infections. Because trypsin cleaves peptides primarily at the C-terminal ends of arginine and lysine, trypsin has been used to cleave influenza virus HA in *in vitro* studies (16, 17). In the final stage of infection, NA facilitates progeny virus release from infected cells by removing sialic acid from the host cell glycoconjugates (18).

Viral–bacterial interactions can enhance the pathogenesis of the respiratory tract (19). Numerous studies have reported that interactions between influenza virus and bacteria influence the pathogenesis of infections (20). Influenza virus infection enhances adhesion of both *Streptococcus pneumoniae* and *Hemophilus influenzae* to respiratory epithelial cells (21). In addition, proteases of certain bacteria, including *Staphylococcus aureus* and *Aerococcus viridans*, can contribute to the activation of HA cleavage and enhance the ability of influenza to undergo efficient viral replication (11, 22, 23, 24). Therefore, it is important to understand the interaction between the influenza virus and bacteria to elucidate the pathogenesis of respiratory infections and to develop strategies to prevent infections.

Periodontal disease is an infectious and inflammatory condition that is one of the most prevalent diseases worldwide. Moreover, periodontal disease results in the destruction of periodontal tissues (including periodontal bone), leading to tooth loss (25). *Porphyromonas gingivalis*, a gram-negative anaerobe, is the most important periodontopathic bacterium that is detectable in the saliva of patients as periodontal disease progresses (26). *P. gingivalis* possesses several virulence factors, including lipopolysaccharide (LPS), fimbriae, and trypsin-like proteases, known as gingipains, which are arginine-specific gingipains (Rgp) that cleave arginine residues, and lysine-specific gingipain (Kgp) that cleave lysine residues (27). In addition to being the major etiological agent of periodontal disease, *P. gingivalis* may be associated with several systemic diseases, such as respiratory disease, diabetes, and atherosclerosis (28, 29).

A recent study reported that subjects with poor oral hygiene are at higher risk of influenza virus infection than those with good oral hygiene (30). Okuda *et al.* reported that professional oral care reduces oral bacteria, resulting in the prevention of influenza viral infections among the elderly (31). Moreover, several clinical studies have reported that *P. gingivalis* is involved in influenza virus infection, wherein *P. gingivalis* was observed at high rates in the lungs of a patient infected with influenza A virus (32), and patients with severe influenza had an increased abundance of the *Porphyromonas* genus in the nasopharynx (33). Thus, although *P. gingivalis* may be associated with influenza viral infections, the underlying molecular mechanisms have not yet been elucidated.

In the present study, we examined the effects of *P. gingivalis* on influenza A virus infections. Our results suggest that Rgp secreted by *P. gingivalis* is capable of cleaving influenza virus HA, thereby increasing viral titers and spreading.

## Results

### *P. gingivalis* culture supernatant affects progeny virus release

**Figure 1:**
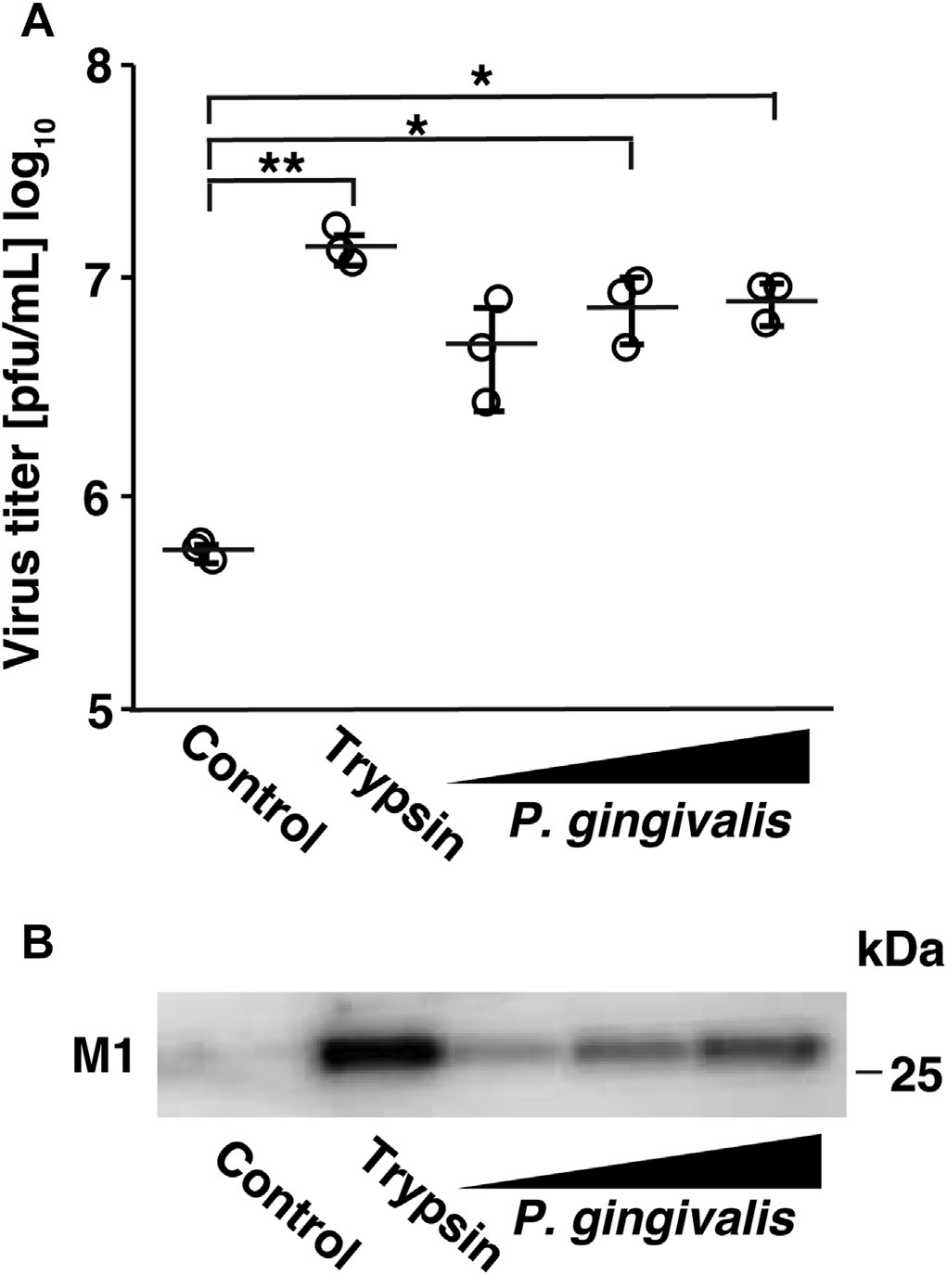
.***Porphyromonas gingivalis* culture supernatant affects progeny virus release.***A*, MDCK cells were inoculated with influenza A/Udorn/72 virus at an MOI of 0.001. After viral adsorption for 30 min, the cells were incubated with *P. gingivalis* FDC381 culture supernatant (0.1, 0.25, or 0.5% v/v). Following 24 h incubation, the culture media were harvested and virus titers were determined using plaque assays. Values are presented as the mean ± SD, *n* = 3. ∗*p* < 0.05; ∗∗*p* < 0.01. The data were analyzed using one-way ANOVA with Tukey’s *post hoc* analysis. *B*, MDCK cells were inoculated with the virus at an MOI of 0.001. After viral adsorption for 30 min, the cells were incubated with *P. gingivalis* FDC381 culture supernatant (0.1, 0.25, or 0.5% v/v). Following 24 h of incubation, the culture supernatants were harvested and viral M1 protein expression was detected using western blotting with a specific monoclonal antibody.

When Madin-Darby canine kidney (MDCK) cells are infected with the influenza virus, trypsin is required to cleave HA for efficient viral infectivity. In this study, to evaluate the potential role of *P. gingivalis* during influenza virus infection, we examined whether viral infectivity was associated with *P. gingivalis* culture supernatant instead of trypsin. First, we analyzed the effects of *P. gingivalis* culture supernatant on progeny virus release. Influenza A/Udorn/72 (H3N2) virus was inoculated into MDCK cells in culture media containing *P. gingivalis* culture supernatant, trypsin, or bacterial culture medium (control). After 24 h of incubation, the culture media were collected and virus titers were determined using plaque assays. When either trypsin or *P. gingivalis* culture supernatant was added to virus-adsorbed cells, the number of progeny viruses released increased markedly (Fig. 1*A*). In addition, the results of the experiment using a human lung cell line (A549 cells) showed that the number of progeny viruses increased in *P. gingivalis* culture supernatants (Fig. S1). However, the cells detached when trypsin was added. Therefore, MDCK cells were used in this study.

Viral M1 proteins line the inner surface of the viral membrane and play a central role in assembly, budding, and morphological processes; thus, determining their expression levels is indicative of virus-infected cells and the amount of virus present in the sample (34). To investigate the effects of *P. gingivalis* culture supernatant on the expression of viral M1 proteins collected from the culture supernatant of infected cells, we performed western blotting using culture media under the same conditions as described in Figure 1*A*. Viral M1 protein expression was not detected in the bacterial culture medium (control), whereas trypsin and *P. gingivalis* culture supernatants significantly induced the expression of these proteins (Fig. 1*B*). These results suggest that substances originating from *P. gingivalis* increase the release of progeny viruses from the infected cells.

### *P. gingivalis* culture supernatant contributes to infectivity and cell-to-cell virus spread

**Figure 2:**
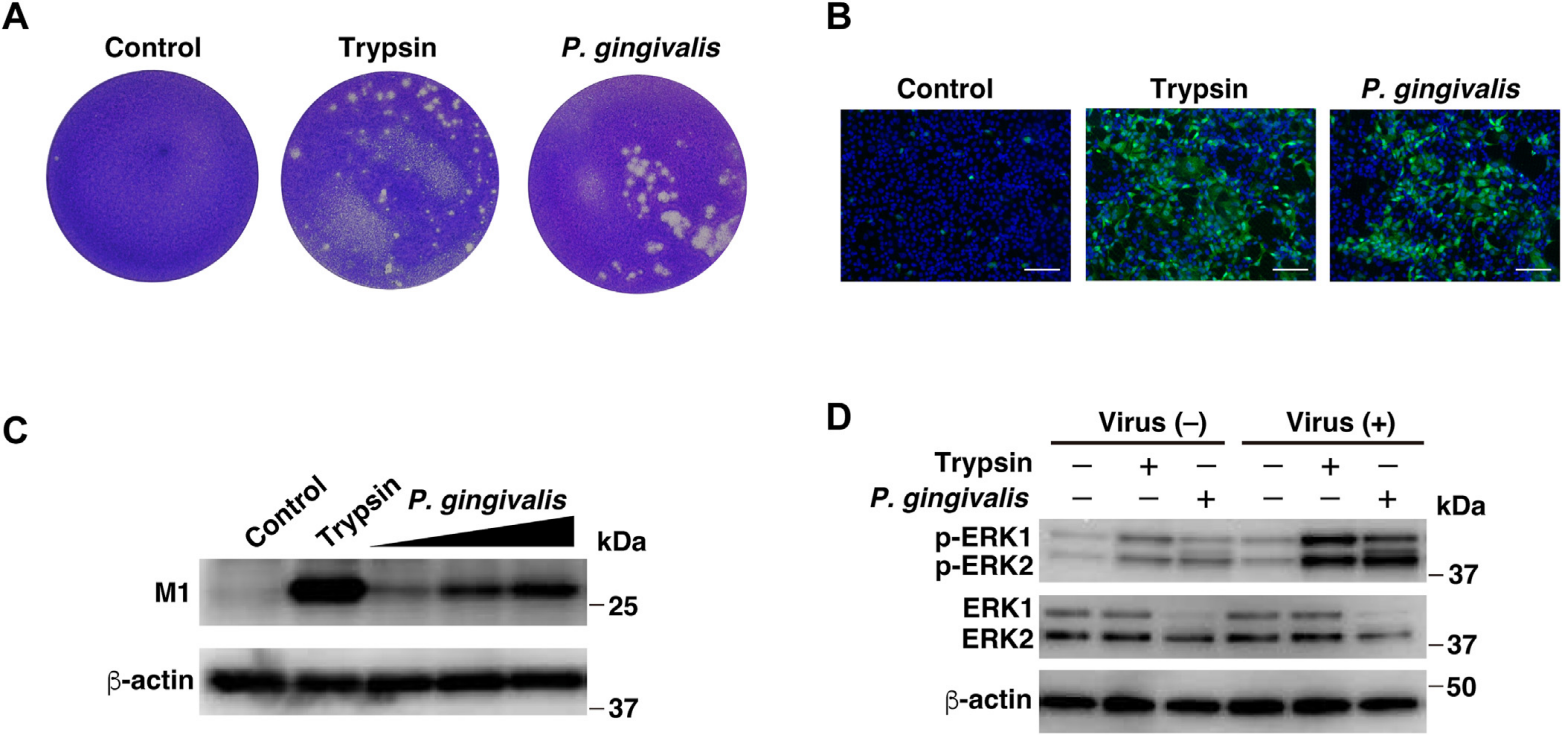
***Porphyromonas gingivalis* culture supernatant contributes to infectivity and cell-to-cell virus spread.***A*, MDCK cells were exposed to 100 pfu/well of influenza A/Udron/72 virus for 30 min and washed. Infected cells were overlaid with 1.6 ml of a solution composed of DMEM containing 0.6% agarose, 1.5% gelatin, and either bacterial culture medium (control), trypsin (1 μg/ml), or *P. gingivalis* FDC381 culture supernatant (0.5% v/v). After incubation for 3 days at 34 °C, plaques were fixed and stained with 0.1% crystal violet solution. *B*, for the indirect IF assay, MDCK cells were inoculated with the virus at an MOI of 0.01. After viral adsorption for 1 h, the cells were incubated for 16 h with either bacterial culture medium (control), trypsin (1 μg/ml), or *P. gingivalis* FDC381 culture supernatant (0.5% v/v). The viral matrix protein was stained with anti-influenza A M1 protein antibody and Alexa Fluor 488 secondary antibody (*green*), and the cell nuclei were stained with Hoechst 33,342 (*blue*). Scale bar, 100 μm. *C*, MDCK cells were inoculated with the virus at an MOI of 0.001. After viral adsorption for 30 min, the cells incubated with bacterial culture medium (control), trypsin (1 μg/ml), or *P. gingivalis* FDC381 culture supernatant (0.1, 0.25, or 0.5% v/v). Following 16 h incubation, cell lysates were harvested and viral M1 protein expression was assessed using western blotting with anti-influenza A M1 protein antibody. β-actin was used as loading control. *D*, *Porphyromonas gingivalis* culture supernatants enhanced ERK1/2 activation in the cells with the virus. MDCK cells were inoculated with or without the virus at an MOI of 0.001 in bacterial culture medium, trypsin (1 μg/ml), or *P. gingivalis* FDC381 culture supernatant (0.5% v/v). After 16 h incubation, cell lysates were harvested and phosphorylated ERK1/2 (p-ERK1/2) or total ERK1/2 were detected using western blotting with specific antibodies. β-actin was used as a loading control.

To evaluate the effect of *P. gingivalis* culture supernatant on influenza viral replication, as determined through viral plaque formation, MDCK cells were inoculated with the A/Udorn/72 virus and overlaid with a culture medium containing agarose, gelatin, and *P. gingivalis* culture supernatant. Viral plaques were detected in the presence of either trypsin or *P. gingivalis* culture supernatant, whereas viral plaques were not detected in the bacterial culture medium (control) (Fig. 2*A*).

Next, we investigated whether the *P. gingivalis* culture supernatant affected the cell-to-cell spread of the infection using immunofluorescence (IF) with an anti-viral M1 protein antibody. MDCK cells were cultured on glass coverslips, inoculated with the A/Udorn/72virus, and incubated in a culture medium containing *P. gingivalis* culture supernatant, trypsin, or bacterial culture medium (control). IF microscopy showed a high degree of cell-to-cell spread of infection upon the addition of *P. gingivalis* culture supernatant, which was similar to trypsin addition (Fig. 2*B*). To further confirm the effects of *P. gingivalis* culture supernatant on the cell-to-cell spread of infection, we detected M1 protein expression in cell lysates using western blotting. We observed that although viral M1 protein expression was not detected in the bacterial culture medium (control), the *P. gingivalis* culture supernatant greatly induced protein expression in a dose-dependent manner (Fig. 2*C*).

The activation of the Raf/MEK/ERK signaling cascade is indispensable for influenza viral replication (35). Therefore, to examine the effects of *P. gingivalis* culture supernatant on viral replication, we examined the effects of *P. gingivalis* culture supernatant on ERK activation among virus-adsorbed cells. When virus-adsorbed cells were incubated in a culture medium containing either *P. gingivalis* culture supernatant or trypsin, an increase in ERK phosphorylation was observed (Fig. 2*D*). Thus, *P. gingivalis* culture supernatant has the potential to support multicycle viral replication.

### *P. gingivalis* culture supernatant influences influenza virus HA cleavage activation

**Figure 3:**
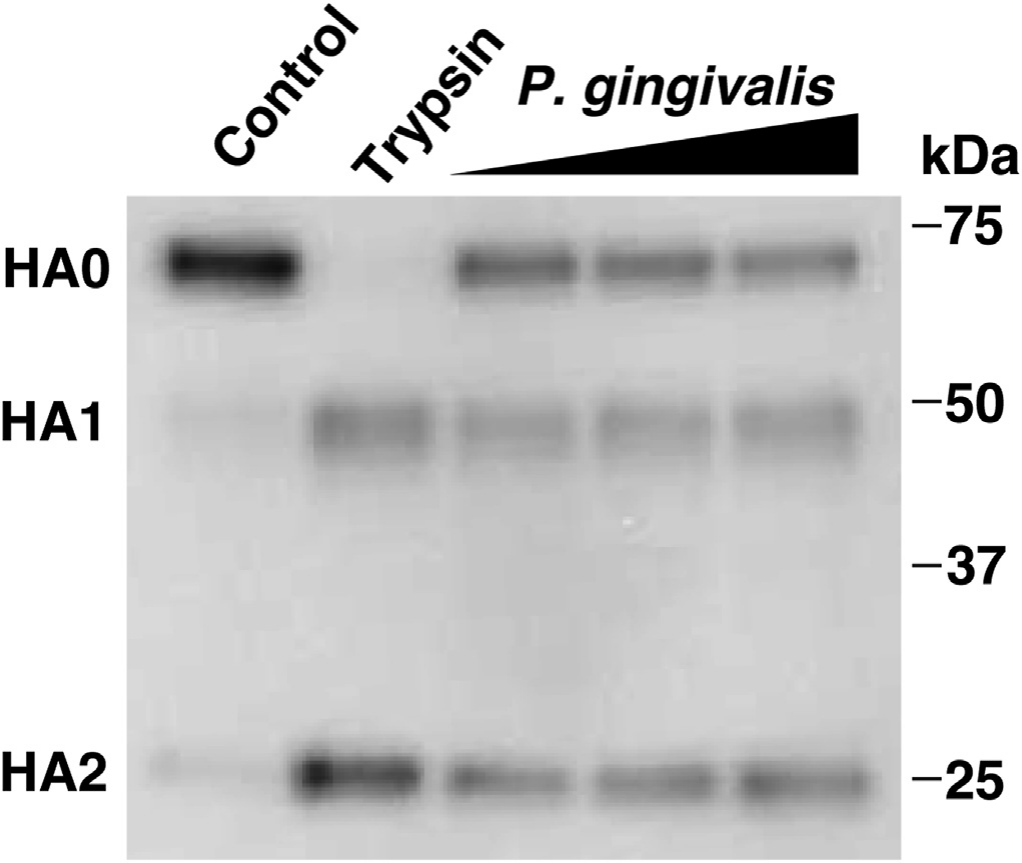
.***Porphyromonas gingivalis* culture supernatant influences influenza virus HA cleavage activation.** Influenza A/Udorn/72 virus was treated with either bacterial culture medium (control), trypsin (1 μg/ml), or *P. gingivalis* FDC381 culture supernatants (0.5, 1, or 2% v/v) at 37 °C for 120 min. HA cleavage activity was assessed using western blotting.

HA cleavage is necessary for viral infectivity because it activates the membrane fusion potential of HA (11). Since *P. gingivalis* culture supernatant can increase the release of progeny viruses and support multicycle viral replication in the absence of trypsin, *P. gingivalis*-secreted protease is considered to have the ability to cleave HA. Therefore, to assess whether *P. gingivalis* culture supernatant can cleave HA, western blotting was performed to analyze putative HA cleavage when A/Udorn/72 virus particles were treated with *P. gingivalis* culture supernatant. As shown in Figure 3, trypsin cleaved HA0 to form HA1 and HA2. Similar results were obtained for the *P. gingivalis* culture supernatant. These results indicated that the trypsin-like protease secreted by *P. gingivalis* has the potential for proteolytic cleavage of HA.

### Gingipain inhibitors hinder cleavage activation of influenza virus HA induced by *P. gingivalis* culture supernatant

**Figure 4:**
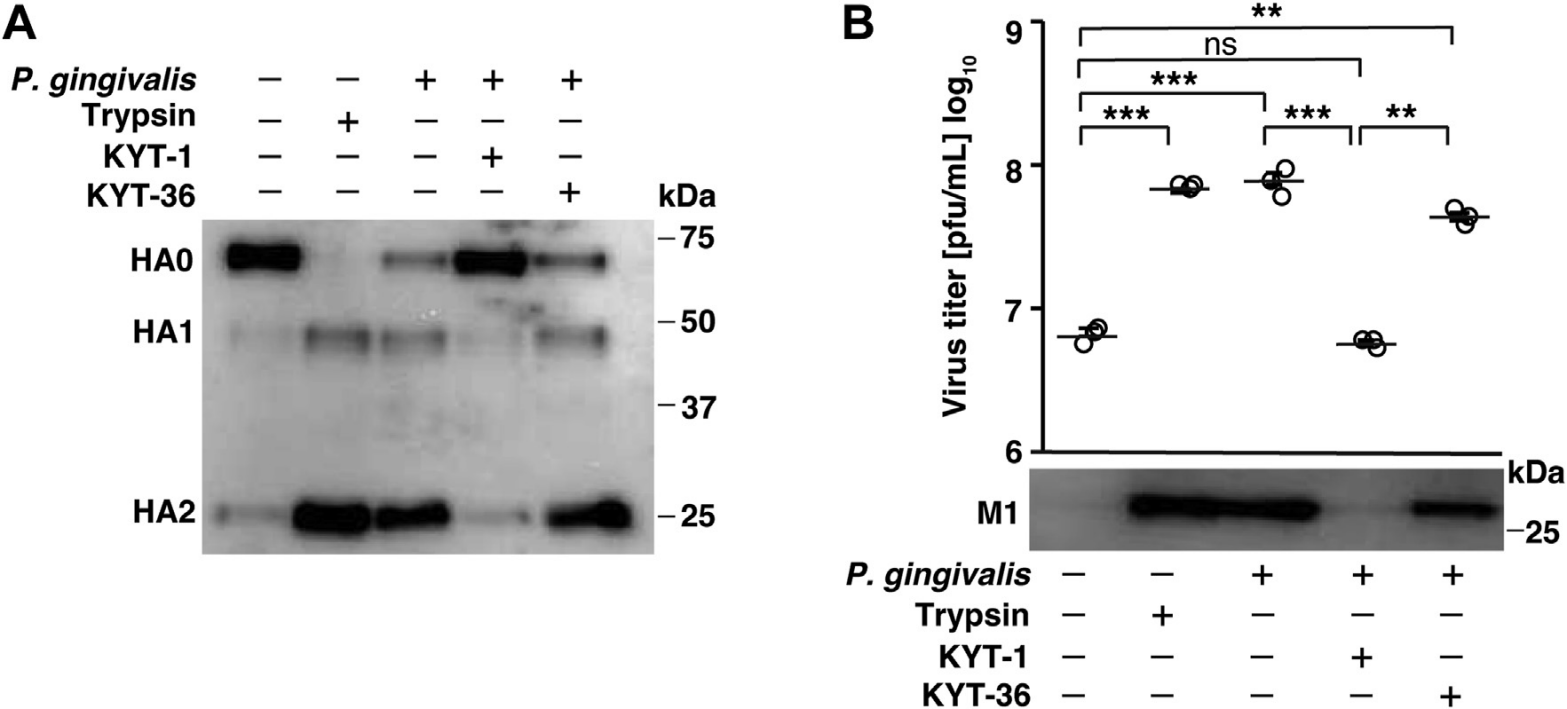
.**Gingipain inhibitors hinders cleavage activation of influenza virus HA induced by *P. gingivalis* culture supernatant**. *A*, Influenza A/Udorn/72 virus were treated with *P. gingivalis* FDC381 culture supernatant (2% v/v) and 5 μM gingipain inhibitor (KYT-1; Rgp inhibitor, KYT-36; Kgp inhibitor) for 120 min. HA cleavage activity was assessed using western blotting. *B*, MDCK cells were inoculated with the virus at an MOI of 0.001. After viral adsorption for 30 min, the cells were incubated in MEM containing either bacterial culture medium, trypsin (1 μg/ml), or *P. gingivalis* FDC381 culture supernatant (0.5% v/v) with or without 5 μM gingipain inhibitors (KYT-1, KYT-36). Following 24 h incubation, the culture media were harvested and virus titers were determined using plaque assays (*upper panel*). Values are presented as the mean ± SD, *n* = 3. ∗∗*p* < 0.01; ∗∗∗*p* < 0.001; ns, not significant. The data were analyzed using one-way ANOVA with Tukey’s *post hoc* analysis. In addition, viral M1 protein expression was detected in the culture media using western blotting with a specific monoclonal antibody (*lower panel*).

*P. gingivalis* produces gingipains with trypsin-like activity. Gingipains are cysteine proteases that include arginine-(Arg-gingipain, Rgp) and lysine-specific gingipains (Lys-gingipain, Kgp) (27). Rgp and Kgp are present in large quantities in *P. gingivalis* culture supernatants (36). We hypothesized that gingipains could be responsible for the proteolytic activation of the influenza virus. To elucidate the role of gingipains in influenza virus infection, we examined the effect of gingipain inhibitors, Rgp inhibitor (KYT-1) and Kgp inhibitor (KYT-36), on the cleavage activation of influenza virus HA by *P. gingivalis* culture supernatant. As shown in Figure 4*A*, KYT-1 inhibited HA cleavage in *P. gingivalis* culture supernatant; however, this effect was not observed with KYT-36. Virus titers measured using plaque assays showed that KYT-1 reduced *P. gingivalis* culture-promoted progeny virus release, whereas virus release was unaffected by KYT-36 (Fig. 4*B*, upper panel). In addition, *P. gingivalis* culture supernatant-induced viral M1 protein was significantly reduced by KYT-1, however, this protein was unaffected by KYT-36 (Fig. 4*B*, lower panel). These results suggest that Rgp activates influenza virus infectivity through the proteolytic cleavage of HA.

### Influenza virus cleavage activation varies among gingipain-deficient *P. gingivalis* mutants

**Figure 5:**
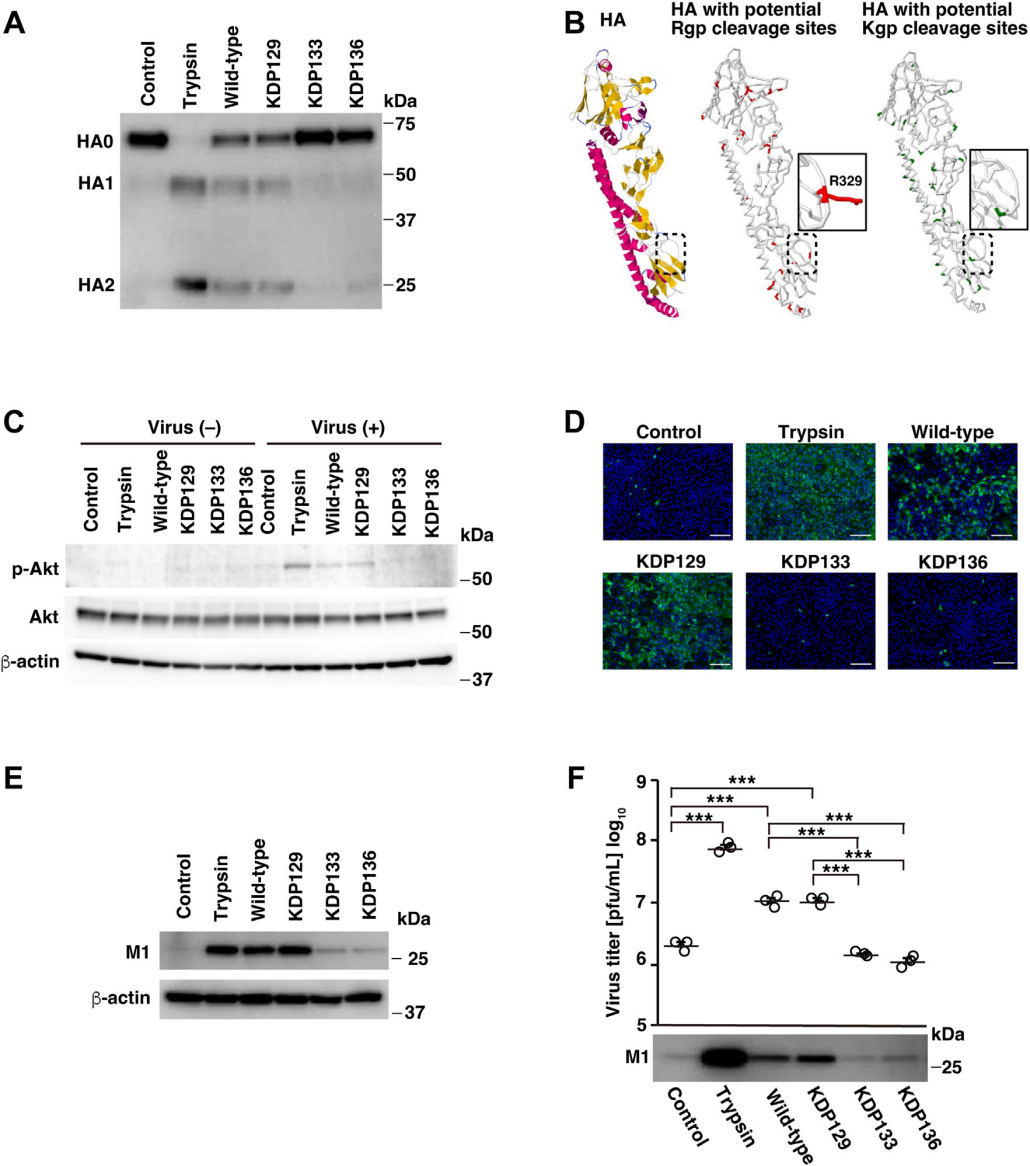
.**Influenza virus cleavage activation varies among gingipain-deficient *P. gingivalis* mutants**. *A*, A/Udorn/72 influenza virus was treated with the culture supernatants (2% v/v) from *P. gingivalis* ATCC 33277 (wild type) or specific gingipain-deficient mutants of *P. gingivalis*, KDP 129 (Δ*kgp*), KDP133 (Δ*rgpA*Δ*rgpB*), and KDP136 (Δ*kgp* Δ*rgpA* Δ*rgpB*) at 37 °C for 120 min. HA cleavage activity was assessed using western blotting. *B*, (*left panel*) generated 1972 H3N2 HA model with potential (*middle panel*) Rgp and (*right panel*) Kgp cleavage sites are shown. HA0 cleavage site is indicated with a dotted box. Potential Rgp cleavage sites are highlighted in *red*; potential Kgp cleavage sites are highlighted in *green*. Magnified view of the HA0 cleavage site is indicated with a *solid box*. R329 involved in the HA0 cleavage site is shown and distinguished in a wireframe display. *C*, MDCK cells were inoculated with or without influenza A/Udorn/72 virus at an MOI of 5. After viral adsorption for 30 min, the cells were incubated in either bacterial culture medium, trypsin (1 μg/ml), or *P. gingivalis* (wild type, KDP129, KDP133, and KDP136) culture supernatant (0.5% v/v). After 4 h of incubation, cell lysates were harvested and phosphorylated Akt (p-Akt) or total Akt were detected using Western blotting using specific antibodies. β-actin was used as loading control. *D*, for the indirect IF assay, MDCK cells were inoculated with the virus at an MOI of 0.01. After viral adsorption for 1 h, the cells were incubated for 16 h in MEM containing trypsin (1 μg/ml) or *P. gingivalis* (wild type, KDP129, KDP133, and KDP136) culture supernatant (0.5% v/v). Viral M1 protein was stained with anti-influenza A M1 protein antibody and Alexa Fluor 488 secondary antibody (*green*), and the cell nuclei were stained with Hoechst 33,342 (*blue*). Scale bar, 100 μm. *E*, MDCK cells were inoculated with the virus at an MOI of 0.001. After viral adsorption for 30 min, the cells incubated in MEM containing trypsin (1 μg/ml) or *P. gingivalis* (wild type, KDP129, KDP133, and KDP136) culture supernatant (0.5% v/v). Following 16 h of incubation, cell lysates were harvested, and viral M1 protein expression was detected using western blotting with a specific monoclonal antibody. β-actin was used as loading control. *F*, MDCK cells were infected with the virus at an MOI of 0.001. After viral adsorption for 30 min, the cells incubated in MEM containing trypsin (1 μg/ml) or *P. gingivalis* (wild type, KDP129, KDP133, and KDP136) culture supernatant (0.5% v/v). Following 24 h incubation, the culture media were harvested, and virus titers were determined by plaque assays (*upper panel*). Values are presented as the mean ± SD, *n* = 3. ∗∗∗*p* < 0.001. The data were analyzed using one-way ANOVA with Tukey’s *post hoc* analysis. Moreover, viral M1 protein expression was detected in the culture media using western blotting with a specific monoclonal antibody (*lower panel*).

Rgp is encoded by both *rgpA* and *rgpB* (37), and Kgp is encoded by the *kgp* gene (38). To confirm Rgp function in influenza virus infectivity, we examined the effects of Kgp-deficient mutant (KDP129: Δ*kgp*), Rgp-deficient mutant (KDP133: Δ*rgpA* Δ*rgpB*), and Kgp/Rgp-deficient mutant (KDP136: Δ*kgp* Δ*rgpA* Δ*rgpB*) culture supernatants on HA cleavage. We assessed gingipain activity in each culture supernatant and confirmed the absence of gingipain activity in each mutant strain (Table S1). Wild-type and KDP129 culture supernatants cleaved HA0, forming HA1 and HA2 products (Fig. 5*A*). In contrast, HA cleavage was diminished in the culture supernatants from KDP133 or KDP136 compared to that in the wild-type and KDP129 strains. Moreover, between the two *P. gingivalis* gingipains (Rgp and Kgp), a potential Rgp cleavage site was recognized at R329, which coincides with the HA0 cleavage site (Fig. 5*B*) (39). This suggests that Rgp can cleave HA0 at the cleavage site, which is consistent with our results. In contrast, no potential Kgp cleavage site is located within the HA0 cleavage site, which is consistent with our results.

Akt phosphorylation occurs during influenza viral entry into host cells (40). Therefore, we examined the effects of the gingipain-deficient mutant culture supernatants on AKT phosphorylation in A/Udorn/72 virus-infected cells. Akt phosphorylation was measured during the treatment of virus-adsorbed cells with trypsin, wild-type, or KDP129 culture supernatant (Fig. 5*C*). In contrast, phosphorylation levels remained unchanged during the treatment of virus-adsorbed cells with either the KDP133 or KDP136 culture supernatant. These findings indicated that the influenza virus HA cleaved by Rgp enables entry into host cells, thereby resulting in Akt phosphorylation.

IF analysis revealed that the number of antigen-positive cells increased in the presence of either wild-type or KDP129 culture supernatants; however, this effect was not observed in either KDP133 or KDP136 culture supernatants (Fig. 5*D*). Moreover, although the culture supernatants of either wild type or KDP129 notably induced viral M1 protein expression in the cell lysates, both KDP133 and KDP136 culture supernatants failed to induce protein expression (Fig. 5*E*). Furthermore, we observed that influenza virus titers were diminished by culture supernatants from either KDP133 or KDP136 compared to culture supernatants from either wild type or KDP129 (Fig. 5*F*, upper panel). Western blot analysis confirmed that wild type and KDP129 culture supernatants increased viral M1 protein levels (Fig. 5*F*, lower panel). In contrast, no such effect was observed in either the KDP133 or KDP136 culture supernatants. These findings demonstrate that Rgp activates influenza virus infectivity through proteolytic HA cleavage.

## Discussion

*P. gingivalis* has been associated with the development and progression of respiratory diseases such as pneumonia and chronic obstructive pulmonary disease (COPD) (41, 42). *P. gingivalis* has been detected in sputum from patients with pneumonia and in tracheal aspirates from patients with COPD (43, 44). Previously, we reported the mechanisms underlying the effect of *P. gingivalis* on respiratory diseases (45, 46, 47, 48). Moreover, the results of several clinical studies suggest that *P. gingivalis* may be involved in influenza virus infection (32, 33), and recent *in vitro* studies have shown that co-infection with *P. gingivalis* and influenza virus significantly enhances the production of inflammatory cytokines compared to infection with *P. gingivalis* or influenza virus alone (49). However, little is known regarding the mechanism through which *P. gingivalis* affects influenza virus infections. In this study, we attempted to elucidate the effects of *P. gingivalis* on influenza viral infection. Here, we showed that the culture supernatant, including gingipain, can cleave viral HA and activate the infectivity of the influenza virus. These observations suggest that *P. gingivalis* contributes to viral infectivity and enhances its pathogenicity.

Viral–bacterial interactions have been reported to enhance pathogenesis in the respiratory tract (19, 20, 50). During influenza virus infection, co-infection with bacteria contributes significantly to both morbidity and mortality (19, 51, 52, 53). Several mechanisms have been proposed to explain influenza virus-bacterial interactions. Influenza virus infection increases vulnerability to secondary *S. aureus* infection by compromising immune cell function (54) and induces ectopic expression of the endoplasmic reticulum chaperone, enabling *S. pneumoniae* to utilize these proteins for deeper tissue invasion (55). In addition, our previous study revealed that NA-producing *Streptococcus oralis*, which is a predominant constituent of human oral flora, promotes the release of influenza virus and the cell-to-cell spread of the infection (56). Interestingly, in the present study, we determined that the *P. gingivalis* culture supernatant cleaved HA and promoted the release of the influenza virus and the cell-to-cell spread of the infection. Proteolytic cleavage of influenza HA is necessary for entry of the virus into the host cell, suggesting that this stage is critical for viral infectivity. The specific cleavage site is located in a loop that protrudes from the surface of HA0 and specific HA0 cleavage exposes a highly conserved fusion peptide (57). HA0 cleavage by enzymes of different specificities yields an N-terminus of HA2 that is different from the highly conserved N-terminus of HA2, resulting in fusion-incompetent HA and non-infectious viruses (58). In this regard, *P. gingivalis* secreted protease included in the culture supernatant is considered to have the ability to specifically cleave HA and does not cleave sites that would cause the virus to lose its infectivity. Based on previous reports, *S. aureus* and *A. viridans* have the ability to cleave influenza HA, and co-infection with bacteria and influenza virus in mice results in a fatal infection with increased viral titers and enhanced lesions in the lung (22, 23, 24). Our present study, along with these studies, provides evidence that a particular bacterial proteases can specifically cleave viral HA and contribute to influenza severity through efficient viral replication (11, 53).

The identities of the HA-cleaving proteases from *S. aureus* and *A. viridans* are still unclear (53). In the present study, we attempted to identify an HA-cleaving protease from *P. gingivalis*. Specifically, the *P. gingivalis* culture supernatant contained three major virulence factors: LPS, fimbriae, and gingipains (Rgp and Kgp). *P. gingivalis* LPS induces immune responses, such as the secretion of proinflammatory cytokines (27, 59); *P. gingivalis* fimbriae are capable of adhering to host cells and other bacteria, which in turn induce proinflammatory cytokines (27, 48); and *P. gingivalis* gingipains are trypsin-like cysteine proteinase and are responsible for at least 85% of all proteases secreted by *P. gingivalis* (60). There are two types of Rgp: RgpA, which consists of catalytic and adhesion domains, and RgpB, which consists of only the catalytic domain. Kgp has one type that consists of both catalytic and adhesion domains. We determined that *P. gingivalis* culture supernatant with an Rgp inhibitor or Rgp-deficient *Porphyromonas ginigivalis* culture supernatant did not cause viral spread and increased viral release. Furthermore, we found that in influenza virus-adsorbed cells, wild-type and Kgp-deficient *P. gingivalis* culture supernatants increased Akt phosphorylation, indicating the entry of the virus into host cells, whereas Akt phosphorylation was unchanged by Rgp-deficient *P. ginigivalis* culture supernatants. Therefore, we propose that Rgp can cleave HA, which allows its entry into host cells.

Rgp and Kgp secreted into *P. gingivalis* culture supernatants exist only in the catalytic domain (61, 62, 63). Moreover, the adhesin domains of RgpA and Kgp are highly homologous (64, 65). Hence, the adhesin domain may not be involved in influenza virus infection. The Rgp and Kgp catalytic domains have specific cleavage sites: Rgp cleaves the Arg-X peptide bond, whereas Kgp cleaves the Lys-X peptide bond (63). Considering that HA0 is cleaved at a specific Arg residue, resulting in HA1 and HA2 (11, 53), these findings provide evidence that the catalytic Rgp domain is capable of specific proteolytic HA cleavage, thereby contributing to viral infectivity.

Notably, additional investigation using human respiratory cell lines and mice are required for the investigation of the effect of *P. gingivalis* on influenza virus infection, this study only used canine kidney cell line, MDCK cells. However, because MDCK cells lack a specific protease for HA cleavage (66), MDCK cells are suitable for the analysis of *P. gingivalis* proteolytic activity. Therefore, our data provide useful insights into the role of *P. gingivalis*-secreted Rgp in influenza virus infection and pathogenicity.

Influenza is more likely to occur in severe cases among the elderly and children and has a high mortality rate (2, 67, 68). Among the elderly, there is an increased risk of aspiration due to reduced laryngopharyngeal sensitivity, resulting in the impairment of both the cough and swallowing reflexes (69). As the salivary concentration of *P. gingivalis* increases in patients with periodontal disease and poor oral hygiene (26), it is possible that *P. gingivalis*-containing saliva is aspirated into the lower respiratory tracts of elderly individuals. In mice infected with *P. gingivalis* along the periodontium, gingipains were detected in the lungs (70) and, in another study using mice, gingipains were shown to have the ability to injure lungs (71). Moreover, clinical studies have indicated that the mean values of trypsin-like proteases, including gingipains, and their activity in saliva are significantly higher in the periodontitis group than in the healthy periodontium group (72, 73). In addition, periodontal treatment and professional oral care decrease trypsin-like protease activity (such as gingipain activity) in the saliva (73) resulting in a decrease in the risk of influenza virus infection (31). These observations indicated that saliva from the periodontitis group enhanced *P. gingivalis*-secreted Rgp activity owing to periodontal disease and poor oral hygiene, which in turn was aspirated into the respiratory tract, leading to an increase in the risk of influenza virus infection and viral pathogenicity.

Influenza vaccination aims to protect high-risk groups from severe cases (especially mortality) and is recommended for these groups (67). The viral antigens used in vaccines are periodically updated because of antigenic drift among circulating influenza viruses. Vaccine effectiveness is evaluated each influenza season to assess performance and WHO makes recommendations on vaccine composition (3). At present, this is the best program and policy planning for vaccine development. However, vaccine effectiveness against H3N2 is extremely low among certain groups like the elderly population (74, 75). This finding highlights the need for the development of various influenza prevention methods. Considering, HA cleavage is the key factor in the early stages of infection; therefore, maintaining healthy oral conditions would result in low Rgp activity, and suppressing this stage may be a promising approach for both preventing and worsening influenza virus infection.

## Experimental procedures

### Reagents

Crystal violet was purchased from Wako Pure Chemical Industries. KYT-1 and KYT-36 were purchased from Peptide Institute.

### Cell culture

MDCK cells were purchased from the American Type Culture Collection (ATCC). MDCK cells were maintained in Minimum Essential Medium (MEM; Thermo Fisher Scientific) supplemented with 10% fetal bovine serum (FBS; Serana Europe GmbH) at 37 °C and 5% CO2. MDCK cells were tested and confirmed to be free of *Mycoplasma* contamination.

### Virus preparation

Influenza A/Udorn/307/72 (H3N2) viral stocks were propagated in MDCK cells and grown at 34 °C and 5% CO_2_ for 40 h in MEM containing 2.5 μg/ml TPCK-trypsin (Worthington Biochemical), penicillin, and streptomycin (Thermo Fisher Scientific). Culture supernatants were collected and supplemented with 0.2% bovine serum albumin (BSA) and subsequently stored at −80 °C until use.

### Uncleaved virus preparations

MDCK cells were infected with A/Udorn/307/72 at a multiplicity of infection (MOI) of two. Viruses were allowed to replicate for 24 h in cells cultured in an incomplete medium without TPCK-trypsin. Culture supernatants were collected and supplemented with 0.2% BSA, and then stored at −80 °C until use.

### Bacterial strains and culture conditions

*P. gingivalis* strains used were FDC381 and ATCC33277 along with isogenic deletion mutants that fail to express Kgp (KDP129; Δ*kgp*) (76), Rgp (KDP133; Δ*rgpA* Δ*rgpB*), and both gingipains (KDP136; Δ*kgp* Δ*rgpA* Δ*rgpB*) (77). ATCC 33277-derived mutant strains were provided by Professor Nakayama (Nagasaki University). *P. gingivalis* was grown in Gifu anaerobic medium (GAM) broth (Nissui) containing 5 μg/ml hemin and 0.5 μg/ml menadione in an anaerobic system (10% CO_2_, 10% H_2_ and 80% N_2_ at 37 °C using TE-HER ANAEROBOX, Hirasawa) for 48 h (FDC381). ATCC33277 and gingipain-deficient mutants were grown until the late log phase. The cultures were clarified through centrifugation (7000*g* for 10 min at 4 °C) and filtered through sterile 0.22-μm-pore polyvinylidene difluoride (PVDF) syringe filters (Millipore).

### Measurement of virus titers

Viral samples were prepared in Hanks’ balanced salt solution (HBSS; Thermo Fisher Scientific) and inoculated onto MDCK cell monolayers in six-well tissue culture plates. The viruses were then adsorbed onto the cells for 30 min at room temperature. Subsequently, 1.6 ml L-15 (Thermo Fisher Scientific) containing 0.6% SeaKem ME agarose, 1.5% gelatin, and 2.5 μg/ml TPCK-trypsin was added to each well and allowed to solidify. Plates were incubated for 3 days at 34 °C, and the number of plaques was then counted. Virus titers were expressed as plaque-forming units (pfu)/ml.

### Western blotting

Culture supernatants and cell lysates were separated using sodium dodecyl sulfate-polyacrylamide gel electrophoresis (SDS-PAGE) and transferred to PVDF membranes (Millipore). Membranes were blocked with 2% BSA and sequentially incubated with primary and secondary antibodies, followed by thorough washing. Primary antibodies included anti-Influenza A H3N2 HA antibody (CEDARLANE), anti-Influenza A Matrix protein antibody (AbDSerotec), anti-ERK1/2 phospho-specific antibody, anti-ERK1/2 antibody, anti-Akt phosphor-specific antibody, anti-Akt antibody (Cell Signaling Technology), and anti β-action antibody (Santa Cruz Biotechnology). HRP-linked anti-mouse IgG and anti-rabbit IgG antibodies (Invitrogen) were used as secondary antibodies. All membranes were treated with ECL Prime Detection Reagent (Cytiva) prior to examination. All bands were visualized using the ChemiDoc XRS System (Bio-Rad).

### Plaque assays

MDCK cell monolayers were inoculated with the influenza virus (100 pfu) in six-well tissue culture plates. The viruses were then adsorbed onto the cells for 30 min at room temperature. Thereafter, 1.6 ml of Dulbecco’s modified Eagle medium (DMEM; Thermo Fisher Scientific) containing 0.6% SeaKem ME agarose (Lonza), 1.5% gelatin (Nacalai Tesque), and *P. gingivalis* culture supernatant, TPCK-trypsin (1 μg/ml), or bacterial culture medium was added to each well and allowed to solidify. Plates were incubated for 3 days at 34 °C and then fixed with 4% paraformaldehyde (PFA) for 1 h. To visualize the viral plaques, fixed cells were incubated with 0.1% crystal violet and 20% methanol in phosphate-buffered saline (PBS) and then washed with water.

### Indirect IF assay

MDCK cells grown on glass coverslips were inoculated with the virus at MOI of 0.01 and incubated for 16 h at 34 °C. Cells were fixed with 4% paraformaldehyde for 5 min, followed by incubation with cold methanol for 5 min at room temperature. Fixed cells were incubated for 1 h with a mouse monoclonal antibody against M1 (Takara Bio) in PBS containing 1% BSA, followed by incubation for 1 h with Alexa Fluor 488 anti-mouse IgG (Thermo Fisher Scientific) in PBS containing 1% BSA and Hoechst 33342 (Thermo Fisher Scientific). Coverslips were mounted on glass slides using ProLong Gold antifade reagent (Thermo Fisher Scientific) and examined under a fluorescence microscope (IX-FLA; Olympus). The obtained images were analyzed using InStudio software (Pixera).

### Proteolytic cleavage of viral HA by *P. gingivalis* culture supernatant

Viruses were treated with 0.5 to 2% v/v *P. gingivalis* culture supernatant and incubated for 120 min at 37 °C. Trypsin (1 μg/ml) was used as a positive control. Following incubation, HA cleavage was analyzed by western blotting as described earlier.

### HA model generation and cleavage site scanning

In the absence of an experimentally generated HA structure for the 1972 influenza A H3N2/Udorn/307 strain in the RCSB database, we generated our own HA model using the amino acid sequence (P19106) found in UniProt (www.uniprot.org) and AlphaFold2 (34265844) (78). Subsequently, we scanned for possible Rgp (R-X-X/R-X-X-X) and Kgp (K-X-X/K-X-X-X) cleavage sites within the generated HA model and highlighted these sites in red (potential Rgp cleavage sites) and green (potential Kgp cleavage sites).

### Statistical analysis

All experiments were performed at least in triplicate, and mean values ± standard deviation (SD) were calculated. Normal distribution of the data was tested using the Shapiro-Wilk test. Analysis of variance (ANOVA) with a *post hoc* Tukey HSD test was performed to test for statistical significance between experiments using KaleidaGraph (Synergy Software). Statistical significance was set at *p* < 0.05.

## Data availability

All data are contained in this article.

## Supporting information

This article contains supporting information.

## Conflict of interest

The authors declare that they have no conflicts of interest with the contents of this article.
