# Supplementary material for: Porphyromonas gingivalis gingipain potentially activates influenza A virus infectivity through proteolytic cleavage of viral hemagglutinin
Source: J Biol Chem. 2025 Jan 8;301(2):108166. doi: 10.1016/j.jbc.2025.108166 (PMC11834065; doi:10.1016/j.jbc.2025.108166)
Supplement: Supplemental Materials [file mmc1.docx]

**Supporting information**

***Porphyromonas gingivalis* gingipain potentially activates influenza A virus infectivity through proteolytic cleavage of the viral hemagglutinin**

Noriaki Kamio^*^, Marni E. Cueno, Asako Takagi, Kenichi Imai^*^

Department of Microbiology and Immunology, Nihon University School of Dentistry, Tokyo 101-8310, Japan

*Correspondence: Noriaki Kamio: kamio.noriaki@nihon-u.ac.jp; Kenichi Imai: imai.kenichi@nihon-u.ac.jp


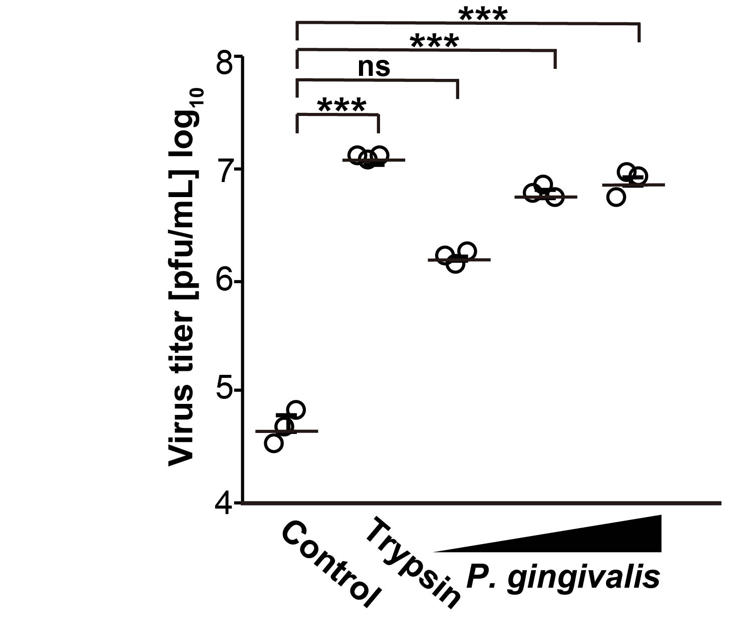
**Figure S1**

**Figure S1 *P. gingivalis* culture supernatant affects progeny virus release in A549 cells.**

A549 cells were inoculated with influenza A/Udorn/72 virus at MOI of 0.01. After viral adsorption for 30 min, the cells were incubated with *P. gingivalis* FDC381 culture supernatant (0.1, 0.25, or 0.5%v/v) or trypsin (0.5 µg/mL). Following 24 h incubation, the culture media were harvested, and virus titers were determined by plaque assays. Values are presented as the mean ± SD, *n* = 3. ****P*<0.001; ns, not significant. The data were analyzed using one-way ANOVA with Tukey´s *post hoc* analysis.

**Table S1** Gingipain activity in gingipain-deficient mutant

|  | Rgp activity | Kgp activity | | |
| --- | --- | --- | --- | --- |
|  | (nmol AMC/min/mL) | | |  |
| Wild-type | 421.0 | 173.1 |  |  |
| KDP129 | 711.6 | 0.1 |  |  |
| KDP133 | 0 | 28.8 |  |  |
| KDP136 | 0 | 0 |  |  |

**Table S1.** Proteolytic activities of Rgp and Kgp in *P. gingivalis* culture supernatants were detected by using 100 µM synthetic substrate, Z-Phe-Arg-MCA for Rgp and Z-His-Glu-Lys-MCA for Kgp, in 20 mM Tris-HCl buffer (pH 7.5) containing 10 mM cysteine and 5 mM CaCl_2_. After incubation at 37 ºC for 30 min, the reaction was terminated by adding 10 mM acetic acid, the released 7-amino-4-methylcoumarin (AMC) was measured at 460 nm (excitation at 355 nm).
